# Introduction to the Special Issue

**DOI:** 10.21542/gcsp.2018.34

**Published:** 2018-08-12

**Authors:** Magdi Yacoub, Robert O Bonow

**Affiliations:** 1 Imperial College, London, UK; 2 Feinberg School of Medicine, Northwestern University, Chicago, USA

The Journal continues its policy of presenting to its readership the latest progress in the fields of science and practice of cardiology in an easily readable and timely manner.

The concept of devoting an issue of the Journal to one topical subject, was recently started to present virtually all aspects of a chosen topic by experts in the field, presenting the latest progress of knowledge in their area.

In the current issue, HCM was chosen because of the recent sustained increase in data relating to both management of, and research into, this relatively common inherited disease which could be of interest to a wide range of our readership.

The current issue represents the proceedings of a recent meeting organised by the Guest Editors in Barcelona. We are grateful to all of them for making this project a reality, and hope that the issue of the Journal will achieve its declared objectives.

